# Acrylamide Exposure Exacerbates Type 2 Diabetes-Induced Neurotoxicity: An Integrated Neurobehavioral and Molecular Investigation

**DOI:** 10.3390/life16030491

**Published:** 2026-03-17

**Authors:** Abdulaziz Arif A. Alshammari, Abdullah Saleh Alkhamiss, Minhajul Arfeen, Razan Alawaji, Mai B. Alwesmi, Vasudevan Mani

**Affiliations:** 1 Department of Pharmacology and Toxicology, College of Pharmacy, Qassim University, Buraydah 51452, Saudi Arabia; 2 Department of Pathology, College of Medicine, Qassim University, Buraydah 51452, Saudi Arabia; 3772@qu.edu.sa; 3 Department of Medicinal Chemistry and Pharmacognosy, College of Pharmacy, Qassim University, Buraydah 51452, Saudi Arabia; m.arfeen@qu.edu.sa; 4 King Salman Medical City, Madina Health Cluster, Madinah 42319, Saudi Arabia; razanalawaji@gmail.com; 5 Department of Medical-Surgical Nursing, College of Nursing, Princess Nourah bint Abdulrahman University, Riyadh 11564, Saudi Arabia

**Keywords:** diabetes mellitus, acrylamide, neuroinflammation, oxidative stress, neurodegeneration

## Abstract

Type 2 Diabetes Mellitus (T2DM) is a widespread metabolic disorder that can affect brain health, primarily through the damaging effects of prolonged hyperglycemia. This condition increases oxidative stress (OS), neuroinflammation, and neuroapoptosis, ultimately impairing cognitive function. Acrylamide (ACY), a neurotoxicant formed during high-temperature food processing and present in cigarette smoke, may further aggravate these neurological disturbances. The present experiment examined the exacerbating effects of T2DM and ACY exposure on cognitive function, neurodegeneration, OS, neuroinflammation, and neuroapoptosis in diabetic rats. T2DM was induced via intraperitoneal injections of nicotinamide and streptozotocin, followed by daily oral doses of ACY for a month. Behavioral assessments (EPM, NOR, and Y-maze) evaluated cognitive performance. Brain tissues were analyzed for biochemical markers of neurodegeneration (GSK-3β, AChE, BACE1), OS (MDA, GSH, Catalase), neuroinflammation (NF-κB, TNF-α, PGE2, COX-2), and neuroapoptosis (Bcl-2, Bax, Caspase-3). Immunohistochemistry of Bcl-2, Bcl-6, CD138, and NF assessed structural brain changes. Results indicated that T2DM and ACY exposure significantly increased the incidence of neurological disturbances. Notably, through increased COX-2, PGE2, MDA, Bax, Bcl-6, Caspase-3, and cognitive decline deficits. This study highlights the harmful neurotoxic amplification of T2DM and ACY exposure, emphasizing the importance of public health measures to reduce ACY exposure through dietary and lifestyle changes, particularly among T2DM populations. Further research into neuroprotective strategies and underlying mechanisms is necessary.

## 1. Introduction

### 1.1. Diabetes Mellitus and Cognitive Dysfunction

Diabetes mellitus (DM) is a chronic metabolic disorder that impacts blood glucose regulation, commonly resulting from insulin deficiency, impaired utilization, or a combination of both. These conditions may lead to severe complications affecting the nervous system and vascular structures [1,2]. There is a rapid increase in the number and prevalence of individuals living with DM [3]. As a global health concern, the estimated prevalence of DM is expected to increase to nearly 600 million individuals across all nations by 2035 [3]. T2DM accounts for over 90% of DM cases, making it the predominant form of the disease [4]. The primary causes of T2DM are the insufficient secretion of insulin by pancreatic β-cells and decreased tissue sensitivity to insulin in peripheral regions. These factors lead to a progressive diminution of the body’s insulin production [5]. Notably, epidemiological research has demonstrated a higher prevalence of cognitive deficits and an increased risk of dementia among individuals with diabetes compared to non-diabetic individuals. Cognitive impairment in patients with diabetes mellitus is recognized as a significant clinical issue affecting both patients and healthcare systems [6]. A previous study reported that approximately 45% of patients with T2DM develop mild cognitive dysfunction [7]. Individuals with T2DM have up to a three times higher likelihood of advancing from mild cognitive decline to dementia [8]. However, the specific pathological mechanisms underlying cognitive dysfunction related to DM are still not clearly explained [9].

### 1.2. Acrylamide: Sources, Formation, Metabolism, and Neurotoxicity

Acrylamide (ACY), with the chemical formula C_3_H_5_NO, is a colorless and odorless crystalline substance that readily dissolves in water [10]. It is acknowledged as a neurotoxin that adversely affects the brain and nerve tissues in mammals, including humans, primarily by disrupting neurotransmitter signaling. It plays a role in various neuropathological changes, such as oxidative stress (OS), impaired intraneuronal transport, heightened neuroapoptosis, deterioration of both dopaminergic and cholinergic systems, and excessive phosphorylation of Tau protein [11]. Concerns regarding ACY exposure escalated in 2002 following the discovery of its formation in various dishes prepared at temperatures typically exceeding 120 °C and low moisture conditions. This formation is partially attributed to the Maillard reaction, which involves specific amino acids, such as asparagine, and reducing sugars. Alternative pathways and precursors have also been suggested to contribute to ACY formation [12]. On the other hand, three potential mechanisms have been proposed for the formation of ACY in products that contain tobacco: a reversible chemical process between NH_3_ and CH_2_=CHCOOH, the Maillard reaction involving asparagine and reducing sugars, and the generation of ACY from acrolein [13].

**Figure 1:**
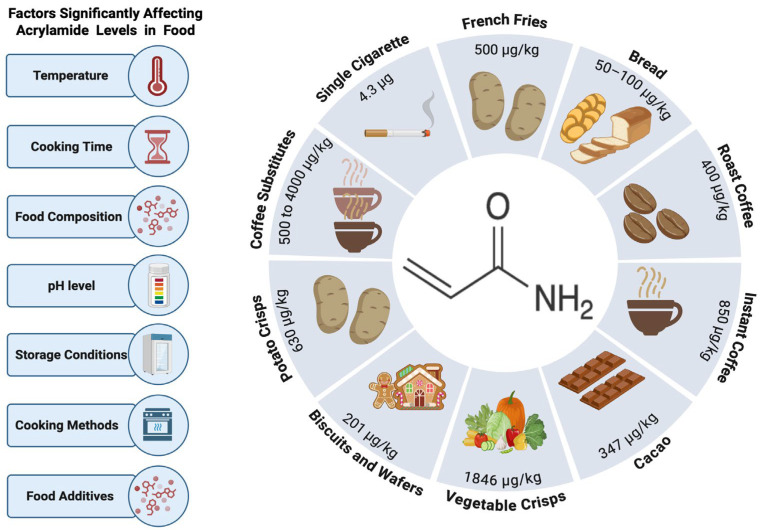
Key factors influencing the formation of acrylamide in foods, accompanied by a comparative overview of acrylamide content (µg) across selected food items and cigarette smoke. Created in BioRender. Alshammari, A. (2026). https://biorender.com/tsj09im (accessed on 6 February 2026).

ACY undergoes metabolism in bodily tissues via two different pathways [14]. Firstly, the P450 enzyme family (CYP2E1) converts ACY into glycidamide, which forms DNA adducts, inhibits neurotransmitter release, and causes nerve damage, highlighting its role in ACY-induced neurotoxicity. Primarily, the second pathway serves to detoxify [14,15]. Research has shown that ACY can cause genotoxic effects during development, neural toxicity, and carcinogenicity effects [16]. In addition, Commission Regulation (EU) 2017/2158 establishes benchmark standards for specific food categories concerning ACY levels. The designated limits are: fried potatoes at 500 μg/kg, bread ranging from 50 to 100 μg/kg, roasted coffee at 400 μg/kg, instant coffee at 850 μg/kg, and coffee substitutes with limits spanning from 500 to 4000 μg/kg [17]. Additionally, commercial potato crisps contain an average ACY concentration of 630 μg/kg [18], while biscuits and wafers contain nearly 200 μg/kg, and vegetable crisps contain 1846 μg/kg [19]. Furthermore, cacao contains 347 μg/kg of ACY [20]. A single cigarette may contain 0.5 to 4.3 μg of ACY [21]. ACY formation in foods is significantly influenced by a number of factors, including temperature, cooking time, food composition, pH level, storage conditions, cooking methods, and the presence of food additives [12] (Figure 1).

### 1.3. Acrylamide and Its Potential Role in DM and Cognitive Impairment

Dietary patterns and smoking are contributing factors for T2DM [13], and major contributors to ACY exposure [22]. Smoking is linked to T2DM because it promotes central obesity, OS, and inflammation, leading to insulin resistance and high blood sugar [13]. Several studies indicate that hyperglycemia triggers and worsens OS and inflammation, contributing to cognitive dysfunction associated with DM [23]. Our previous investigations demonstrated that ACY exposure increases oxidative stress, neuroinflammation, and neuroapoptosis, leading to impaired cognitive performance and structural changes in brain architecture [14,24]. In the general adult population, exposure to ACY has been linked to increased fasting blood glucose, DNA damage, and lipid peroxidation, which together contribute to the ACY-related increase in fasting plasma glucose levels [25]. OS serves a pivotal role in the progressive development and complications of DM [26]. These findings suggest that exposure to ACY may contribute to the development of T2DM [27]. Nutritional exposure to ACY has also been reported to exacerbate diabetic cognitive impairment through mechanisms involving oxidative stress, neuroinflammation, and disruption of key metabolic pathways.

Despite the well-documented neurotoxic effects of ACY and links between T2DM and cognitive dysfunction, the combined impact of ACY in diabetic conditions remains underexplored. T2DM individuals may be more vulnerable to neurotoxic agents due to hyperglycemia, oxidative stress, and inflammation. Studying ACY’s interaction with diabetic pathology could reveal if it worsens neurodegeneration and cognitive issues in diabetes.

### 1.4. Aim of the Study

Given the previously noted health risks of ACY exposure via smoking and dietary consumption, this study aimed to investigate its impact in a nicotinamide hydrochloride (NTH)- and streptozotocin (STZ)-induced rat model of T2DM. We placed key focus on investigating the mechanistic effects of ACY by assessing its ability to augment OS, neurodegeneration, neuroinflammation, and neuroapoptosis, as well as immunohistochemical changes in brain tissue. We examined the effect of exposure to ACY on cognitive function, brain anatomy, fasting blood glucose levels, and body weight in an effort to understand its general systemic and neurologic impact within the diabetic state.

## 2. Materials and Methods

### 2.1. Chemicals

STZ and NTH were acquired from Ambeed, Buffalo Grove, IL, USA. ACY was procured from Sigma-Aldrich, St. Louis, MO, USA. All ELISA kits for rat tissue analysis utilized in this study were obtained from MyBioSource, San Diego, CA, USA, and immunohistochemical antibodies were sourced from Leica Biosystems (Buffalo Grove, IL, USA) and Agilent Dako (Santa Clara, CA, USA).

### 2.2. Animals

The current study was conducted at the Qassim University (QU) Animal Research Facility within the College of Pharmacy. The research received ethical approval from the Research Ethics Committee at QU (Project No. 24-14-03). To promote transparency and reproducibility, the experiment was conducted in accordance with the Animal Research: Reporting of In Vivo Experiments (ARRIVE) 2.0 guidelines, with meticulous adherence to the ARRIVE Essential 10 [28]. The study was conducted with a primary focus on minimizing animal suffering. The experiment employed Sprague Dawley rats, each aged three months and with an average weight of 190 g (±10 g). A total of 24 male rats were involved. The rodents were randomly assigned to four groups, each comprising six rats, and housed in three cages constructed from polymer material under controlled conditions, with an average temperature of 22 °C (±1 °C), an average humidity level of 50% (±5%), and continuous access to food and water.

### 2.3. T2DM Induction

Prior to injection, NTH was dissolved in saline, and STZ was freshly prepared in cold citrate buffer (0.1 M, pH 4.5) immediately before administration. Rats were fasted for 12 h prior to the procedure. Type 2 diabetes mellitus (T2DM) was induced by an intraperitoneal injection of NTH (120 mg/kg), followed 15 min later by STZ (60 mg/kg). Diabetes induction was confirmed three days after injection by measuring fasting blood glucose levels using an Accu-Chek glucometer (Roche, Mannheim, Germany). Rats with fasting blood glucose levels ≥ 127 mg/dL were considered diabetic [29]. Fasting blood glucose levels were subsequently monitored on days 1, 15, and 30 during the experimental period.

### 2.4. Experimental Groups and Treatment Timeline

**Figure 2:**
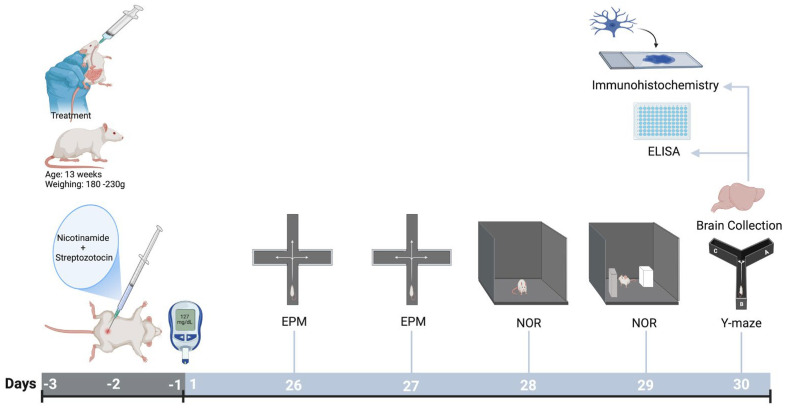
Diabetes was induced using NTH and STZ, confirmed by fasting blood glucose ≥127 mg/dL. Rats were randomly divided into four groups: CON, DIA, ACY, and DIA + ACY (n = 6 each), receiving oral treatments for a month. In the final four days, rats underwent three cognitive tests. After the last test, brain tissues were collected for ELISA and IHC analyses. Created in BioRender. Alshammari, A. (2026). https://biorender.com/emwy9y3 (accessed on 6 February 2026).

After confirmation of T2DM induction, Sprague Dawley rats were randomly assigned to four experimental groups. The control group (CON) received normal saline (NS; 1 mL/kg, p.o.) daily for 30 days. The diabetic group (DIA) consisted of T2DM rats receiving NS (1 mL/kg, p.o.) for the same duration. The ACY group received acrylamide (20 mg/kg, p.o.) once daily for 30 days, while the DIA + ACY group consisted of diabetic rats treated with ACY (20 mg/kg, p.o.) for the same period. ACY was freshly dissolved in NS prior to administration, and the selected dose and route were based on our previous studies [15,27]. Behavioral assessments were conducted during the final four days of the experiment: the Elevated Plus Maze (EPM) acquisition phase on day 26 and retention test on day 27, the Novel Object Recognition (NOR) habituation on day 28, followed by training and testing on day 29, and the Y-maze test on day 30. Immediately after the final behavioral test, brain tissues were collected for ELISA and immunohistochemical (IHC) analyses (Figure 2).

### 2.5. Neurobehavioral Evaluations

#### 2.5.1. EPM

**Figure 3:**
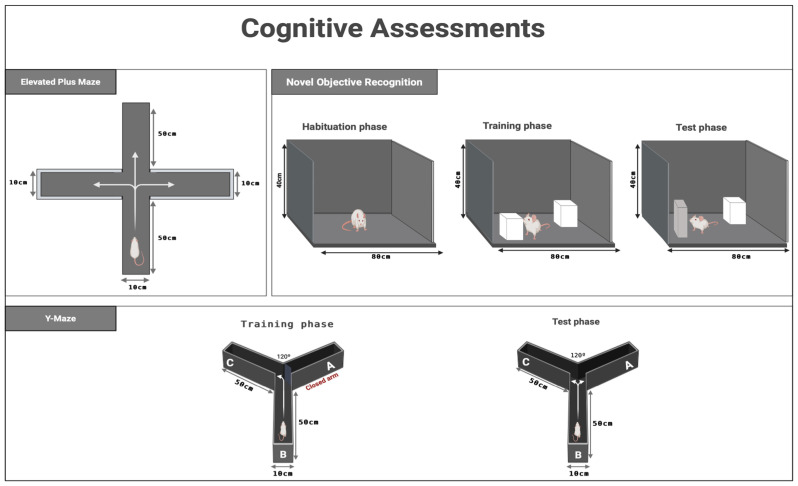
Neurobehavioral tests for rodents include the EPM, NOR, and Y-maze. The EPM assesses learning and memory, and anxiety. The NOR measures recognition memory, and the Y-maze evaluates spatial working memory via spontaneous alternation behavior. Created in BioRender. Alshammari, A. (2026). https://biorender.com/qhvix4x (accessed on 6 February 2026).

To assess the rats’ cognitive abilities, an EPM test was performed. The measure of their performance involved a parameter termed transfer latency (TL). The EPM comprises two open arms, each measuring 50 cm by 10 cm, and two closed arms of identical length and width, but with a height of 40 cm. Constructed from wood, the entire apparatus is elevated 50 cm above the ground. The latency of each rat in transitioning from an open arm to a closed arm was recorded as TL, with an upper cutoff time of 90 s [14]. Besides this, the EPM protocol comprised two distinct phases: acquisition on day 26 and retention on day 27. During the acquisition phase, each rat was individually positioned in the central area of the maze and allowed to explore for two minutes. The same procedure was repeated during the retention phase to assess memory retention. TL on the initial day (day 26) demonstrated the rats’ capacity to learn, whereas the TL on the subsequent day (day 27) indicated their retention of the acquired information (Figure 3).

#### 2.5.2. NOR

The cognitive NOR test is utilized for evaluating memory capabilities [30]. The NOR is a box-like wooden structure measuring 80 cm by 60 cm by 40 cm, with a black coloration. The test serves as a neurobehavioral assessment tool to evaluate recognition memory in rats, comprising two familiarization objects—two white cubes designated as F1 and F2—and one unfamiliar object, a rectangular black item classified as the novel object (N). This behavioral assessment is conducted in three distinct phases: habituation, training, and testing. These phases are carried out over two consecutive days, with each session lasting precisely five minutes. On the first day (day 28), animals undergo exposure to an empty wooden box during the habituation phase. On the subsequent day (day 29), both the training and testing procedures are executed, separated by a four-hour interval. During training, each rat is introduced into a box containing two identical objects (F1 and F2) to facilitate object familiarity. Following this, the test session is conducted in the same apparatus, but F2 is replaced by the N object. The duration of investigation for each object—the novel and the familiar—is recorded. Investigation is defined as a behavior in which the rat brings its nose within 2 cm of an object, makes direct contact, or engages in sniffing. Lastly, a discrimination index (DI%) is computed for each subject to quantify recognition memory, based on the different exploration times between the familiar and the novel object (Figure 3) [14].

#### 2.5.3. Y-Maze

In rodent studies, the Y-maze is employed to evaluate short-term memory, specifically through the measure of spontaneous alternation, which assesses spatial working memory. Rodents are allowed to freely explore the three arms of the maze, driven by their innate curiosity to explore unfamiliar environments [31]. The Y-maze apparatus consists of three arms positioned at 120° angles. Each arm has approximate dimensions of 10 cm in width, 50 cm in length, and 30 cm in height. Within this configuration, arms B and C are designated as familiar arms, whereas arm A serves as the novel arm. This evaluation was conducted over two distinct sessions, each lasting five minutes, on day 30, the final day of the experimental period. During the initial session (training phase), the subjects were placed at the distal end of arm B and permitted to navigate only arms B and C, with access to arm A being restricted. Following a four-hour rest period, the testing session commenced, during which all three arms were accessible. In this phase, the primary focus was on recording the frequency of entries into the familiar (NEKA) versus the novel arm (NENA), as well as the duration spent in each. To evaluate spatial memory and novelty preference, the proportion of time spent in the novel arm (TSNA%) was documented [14] (Figure 3).

### 2.6. Biomolecular Analysis

#### 2.6.1. Tissue Preparation

Following the completion of the final cognitive assessment on day 30, all rodents were humanely euthanized employing an anesthetic protocol consisting of ketamine at 100 mg/kg and xylazine at 10 mg/kg. Immediately after euthanasia, each brain was meticulously extracted and sagittally sectioned. One hemisphere was carefully homogenized in cold phosphate-buffered saline (4 °C) to maintain tissue integrity. The homogenate was subsequently centrifuged at 4000 rpm for 10 min to obtain the supernatant. This supernatant was then rapidly stored at −80 °C for subsequent biochemical analysis. Protein concentrations within these samples were determined using the biuret colorimetric assay. All biomarker measurements obtained from ELISA assays were normalized to the total protein concentration of each brain homogenate sample and expressed as ng/g protein, pg/g protein, or µmol/g protein where appropriate. The remaining hemispheres were placed into individually labeled containers containing 10% buffered formalin in preparation for immunohistochemical analysis.

#### 2.6.2. Molecular Biomarker

To investigate various brain-associated biomarkers, rat-specific ELISA kits were utilized. The protein biomarkers assessed for involvement in neurodegenerative processes included β-Site Amyloid Precursor Protein Cleaving Enzyme-1 (BACE1) (MBS2886958), Glycogen Synthase Kinase-3 Beta (GSK-3β) (MBS284677), and Acetylcholinesterase (AChE) (MBS2709297). Neuroinflammatory mediators examined in this study comprised Nuclear Factor Kappa-B (NF-κB) (MBS453975), Tumor Necrosis Factor-Alpha (TNF-α) (MBS162068), Cyclooxygenase-2 (COX-2) (MBS266603), and Prostaglandin E2 (PGE2) (MBS7606497). Indicators of cell death that were investigated included B-Cell Lymphoma-2 (Bcl-2) (MBS2515143), BCL-2–Associated X Protein (Bax) (MBS165136), and Caspase-3 (MBS018987). Furthermore, oxidative stress was examined through the determination of Malondialdehyde (MDA) (MBS268427), Reduced Glutathione (GSH) (MBS265966), and catalase (MBS2704433) levels.

#### 2.6.3. Immunohistochemistry

To further investigate neuropathological alterations, immunohistochemical analysis was conducted to establish cellular and structural markers of critical importance for neuron survival, inflammation, and axonal health. The regulation of apoptosis was quantified through Bcl-2, an anti-apoptotic marker. Inflammatory pathways were examined via B-Cell Lymphoma-6 (Bcl-6), a transcriptional repressor involved in immune signaling. Cluster of Differentiation 138 (CD138) was employed to assess immune cell infiltration and extracellular matrix remodeling. Neurofilaments (NF), which are essential components of the neuronal cytoskeleton, served as markers of axonal structure and integrity. All staining procedures and reagent concentrations conformed to the recommended protocols provided by the respective manufacturers. Table 1 presents comprehensive details of the immunohistochemical antibodies used in this study, including the stain name, manufacturer, antibody formulation, type, and clone designation.

### 2.7. Statistical Analysis

Data analysis was conducted by calculating mean values and measures of variability. One-way analysis of variance (ANOVA) using GraphPad Prism software (version 9.5.0) was employed to evaluate the comparisons among experimental groups. To further investigate group-wise differences, a Tukey–Kramer post hoc comparison test was performed. Differences were considered statistically significant when the *p*-value was less than or equal to 0.05.

## 3. Results

### 3.1. Impact of DIA and ACY on Fasting Body Weight

**Figure 4:**
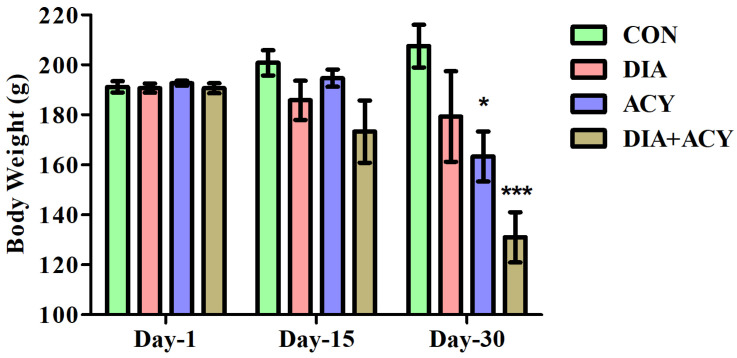
Effects of DIA and ACY treatments on fasting body weight in rats over a 30-day period. Data are presented as mean ± SEM (n = 6 per group). Statistical analysis was performed using one-way ANOVA followed by the Tukey–Kramer post hoc test. Significant comparisons with the CON group (Day-30) are indicated as * *p* < 0.05 and *** *p* < 0.001.

No notable differences were observed among the groups on the first day of testing [Day 1; F(3,20) = 0.2682, *p* = 0.8475] or at the midpoint of the experimental period [Day 15; F(3,20) = 2.239, *p* = 0.1151], as demonstrated by a one-way ANOVA. In contrast, significant differences were shown on the last day of the experimental period [Day-30; F(3,20) = 12.47, *p* < 0.0001], as demonstrated in Figure 4. Following a one-way ANOVA, we utilized the post hoc Tukey–Kramer test for multi-comparison among all experimental groups. Notably, there were no significant variations among all experimental groups in fasting body weight on day 1 and day 15. However, on day 30, there was a remarkable loss in fasting body weight between the ACY alone group (*p* < 0.05) compared to the CON group. Similarly, there was a significant loss in rats’ fasting body weight in the DIA + ACY group (*p* < 0.001) compared to the CON group. The rest of the experimental groups did not exhibit any significant changes on day 30.

### 3.2. Impact of DIA and ACY on Fasting Blood Glucose

**Figure 5:**
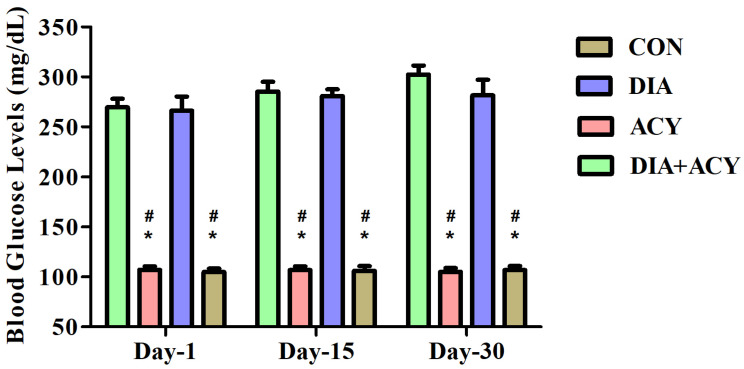
Effects of DIA and ACY exposure on fasting blood glucose levels in rats over a 30-day period. Data are presented as mean ± SEM (n = 6 per group). Statistical analysis was performed using one-way ANOVA followed by the Tukey–Kramer post hoc test. Significance levels are indicated as: * *p* < 0.001 indicates comparisons with the CON group, and # *p* < 0.001 indicates comparisons with the ACY group at the corresponding time points.

Significant fluctuations in fasting blood glucose levels were observed based on one-way ANOVA across all assessment points, namely Day 1 [F(3,20) = 117.5, *p* < 0.0001], the midpoint of the experiment [Day 15; F(3,20) = 233.4, *p* < 0.0001], and the final measurement [Day 30; F(3,20) = 130.4, *p* < 0.0001], as illustrated in Figure 5. To further investigate differences among all treatment groups, Tukey–Kramer post hoc testing was employed. As anticipated, blood glucose levels on Day 1 were significantly lower in the CON group in comparison to both the DIA and DIA + ACY groups (*p* < 0.001). Similarly, the ACY group demonstrated significant differences (*p* < 0.001) when compared with the DIA and DIA + ACY groups. This pattern persisted on Days 15 and 30, with the DIA and DIA + ACY groups showing statistically significant increases in glucose levels relative to the CON group. Nonetheless, no significant differences were observed between the DIA and DIA + ACY groups, nor between the CON and ACY groups across all testing intervals (Days 1, 15, and 30).

### 3.3. Impact of DIA and ACY on the Time Duration for TL in the EPM Test

**Figure 6:**
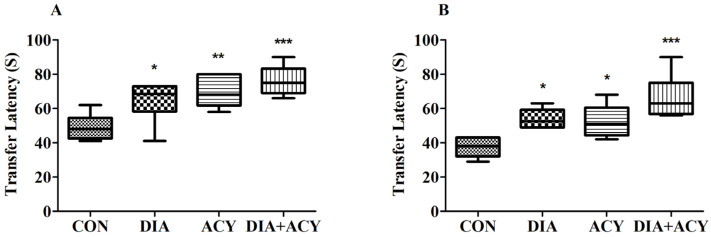
Effects of DIA and ACY treatments on TL duration in the EPM test in rats, assessed on day 1 (**A**) and day 2 (**B**). Data are shown as mean ± SEM (n = 6 per group). One-way ANOVA with Tukey–Kramer post hoc test was used. Significance levels: * *p* < 0.05, ** *p* < 0.01, *** *p* < 0.001 versus CON group.

As previously outlined, the EPM test comprises two distinct phases: the acquisition phase occurring on Day 1 (Day 26 of the treatment schedule) and the retention phase on Day 2 (Day 27 of the treatment schedule). TL, recorded in seconds, served as an indicator of cognitive performance, with shorter durations signifying superior spatial learning and memory. Statistical evaluation using one-way ANOVA revealed significant differences in TL among the groups on both days: Day 1 [F(3,20) = 8.872, *p* = 0.0006] and Day 2 [F(3,20) = 10.60, *p* = 0.0002] (refer to Figure 6). To further delineate the differences between groups, a Tukey–Kramer post hoc comparison was employed. Results from Day 1 (Figure 6A) indicated an increased TL in the treatment groups relative to the CON group (49.00 ± 3.07). Rats in the DIA group exhibited a significantly elevated TL (64.67 ± 4.97, *p* < 0.05), and the ACY group also showed a noteworthy increase (69.50 ± 3.73, *p* < 0.01). The DIA + ACY group demonstrated the longest TL (76.17 ± 3.49, *p* < 0.001), markedly surpassing that of the control group. No other significant differences were observed among the remaining groups.

Consistent trends were observed on Day 2 (Figure 6B), with all treatment groups exhibiting longer TL times in comparison to the CON group. The DIA group (54.00 ± 2.28, *p* < 0.05) and the ACY group (52.50 ± 3.87, *p* < 0.05) both demonstrated significantly prolonged TL durations relative to the CON group (37.33 ± 2.39). The combined treatment group also exhibited a substantial increase in TL (66.50 ± 5.29, *p* < 0.001). Statistical analysis conducted on Day 2 indicated no significant differences among the remaining assessed parameter groups.

### 3.4. Impact of DIA and ACY on Neurobehavioral Performance in the NOR Test

**Figure 7:**
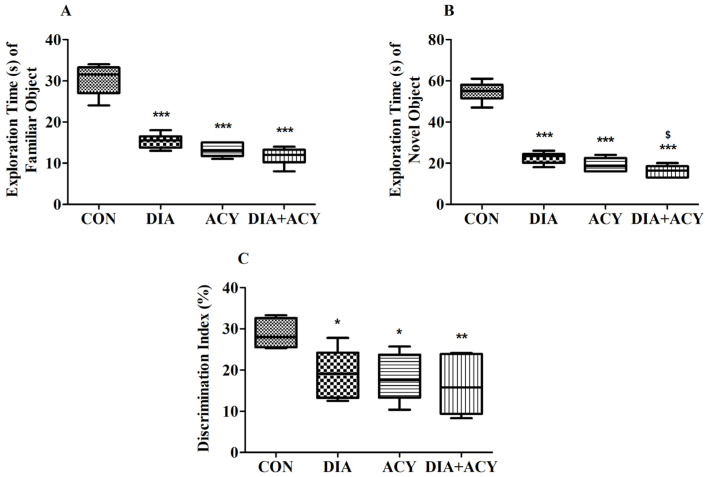
Effects of DIA and ACY treatments on (**A**) ETFO, (**B**) ETNO, and (**C**) DI% during the test phase of the NOR task in rats. Data are shown as mean ± SEM (n = 6 per group). One-way ANOVA with Tukey–Kramer post hoc test was used. Significance levels: * *p* < 0.05, ** *p* < 0.01, *** *p* < 0.001 versus CON group; $ *p* < 0.05 versus DIA group.

The one-way ANOVA results for ETFO (Exploration Time of Familiar Object) demonstrated statistically significant differences among the experimental groups [F(3,20) = 73.53, *p* < 0.0001]. To further investigate these group differences, a Tukey–Kramer post hoc comparison was performed. The ETFO parameter, recorded in seconds, exhibited notable reductions in the treatment groups relative to the CON group (30.33 ± 1.52), as depicted in Figure 7A. Specifically, the DIA group showed a substantially decreased ETFO time (15.33 ± 0.72, *p* < 0.001), followed by more pronounced reductions in the ACY group (13.17 ± 0.65, *p* < 0.001) and the DIA + ACY group (11.67 ± 0.88, *p* < 0.001). Statistical analysis indicated no significant differences among the remaining evaluated parameter groups.

In Figure 7B, the ETNO (Exploration Time of Novel Object) analysis results also revealed statistically significant differences across all groups [F(3,20) = 151.8, *p* < 0.0001]. Post hoc comparisons confirmed substantial reductions in time for all treatment groups relative to the CON group. The DIA group demonstrated a decrease (22.67 ± 1.15, *p* < 0.001) compared to the CON group’s value (54.67 ± 1.91). Similarly, the ACY group (19.17 ± 1.47, *p* < 0.001) and the DIA + ACY group (16.17 ± 1.14, *p* < 0.001) also exhibited significantly shorter ETNO times. Additionally, the DIA + ACY group presented a statistically lower time than the DIA group alone (*p* < 0.05). The remaining groups did not show notable differences in the measured parameters outcomes.

Figure 7C depicts the DI% results, where ANOVA testing demonstrated statistically significant differences across all experimental groups [F(3,20) = 5.545, *p* = 0.0062]. Further analysis using the Tukey–Kramer test revealed that all treatment groups exhibited significantly reduced DI% values in comparison to the CON group (28.76 ± 1.46). Specifically, the DI% levels were considerably lower in the DIA group (19.18 ± 2.36, *p* < 0.05), the ACY group (18.10 ± 2.31, *p* < 0.05), and the DIA + ACY group (16.25 ± 3.08, *p* < 0.01) relative to the CON group. The other groups did not demonstrate any statistically significant differences DI%.

### 3.5. Impact of DIA and ACY on the Neurobehavioral Performance in the Y-Maze Test

**Figure 8:**
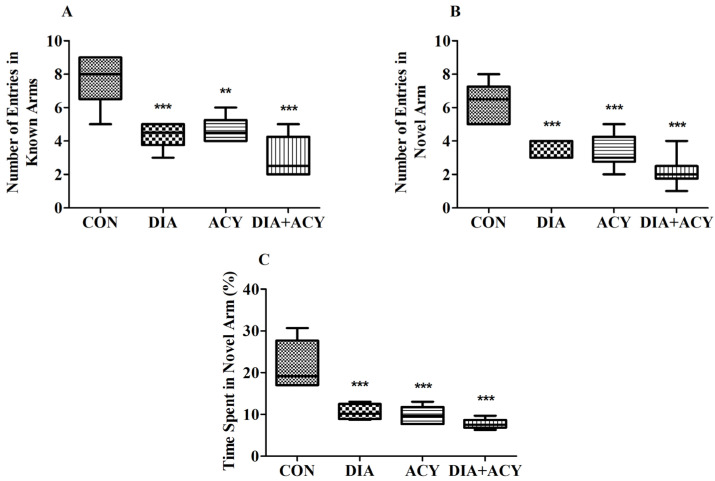
Effects of DIA and ACY treatments on (**A**) NEKA, (**B**) NENA, and (**C**) TSNA% in rats during the Y-maze test. Data are shown as mean ± SEM (n = 6 per group). One-way ANOVA with Tukey–Kramer post hoc test was used. Significance levels: ** *p* < 0.01, *** *p* < 0.001 versus CON group.

Figure 8 summarizes the results of the Y-Maze cognitive task, indicating statistically significant differences among all experimental groups based on one-way ANOVA. The findings were as follows: NEKA [F(3,20) = 17.91, *p* < 0.0001], NENA [F(3,20) = 19.75, *p* < 0.0001], and TSNA% [F(3,20) = 22.41, *p* < 0.0001]. To conduct a more detailed analysis of the group-wise differences, a Tukey–Kramer post hoc test was performed.

In the NEKA assessment (Figure 8A), all treatment groups demonstrated a statistically significant reduction compared to the CON group. The DIA group recorded a lower NEKA value (4.33 ± 0.33, *p* < 0.001) relative to the CON group (7.67 ± 0.62). The ACY-treated group also exhibited a notable decline (4.67 ± 0.33, *p* < 0.01), and a more pronounced reduction was observed in the DIA + ACY group (3.00 ± 0.52, *p* < 0.001). No additional significant changes in NEKA values were identified among the remaining groups.

Similarly, for the NENA parameter (Figure 8B), both the DIA (3.67 ± 0.21, *p* < 0.001) and ACY (3.33 ± 0.42, *p* < 0.001) groups demonstrated significantly lower values compared to the CON group (6.33 ± 0.49). A comparable reduction was also observed in the DIA + ACY group (2.17 ± 0.40, *p* < 0.001). No additional differences among other groups regarding NENA were observed or identified.

Corresponding patterns were observed in the TSNA% results (Figure 8C). The percentage values were markedly lower across all treatment groups, with DIA (10.56 ± 0.75, *p* < 0.001), ACY (9.83 ± 0.87, *p* < 0.001), and DIA + ACY (7.72 ± 0.48, *p* < 0.001) each demonstrating statistically significant reductions relative to the control group (21.61 ± 2.33). TSNA% was expressed as a percentage. No other significant differences in TSNA% were identified among the remaining experimental condition groups.

### 3.6. Impact of DIA and ACY on Specific Neurodegenerative Markers in the Rat Brain

**Figure 9:**
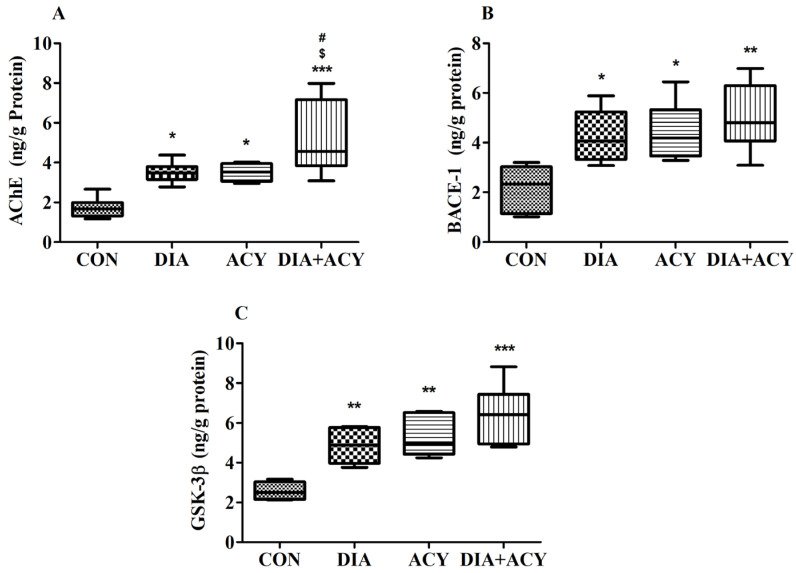
Effects of DIA and ACY treatments on (**A**) AChE, (**B**) BACE-1, and (**C**) GSK-3β in rats. Data are shown as mean ± SEM (n = 6 per group). One-way ANOVA with Tukey–Kramer post hoc test was used. Significance levels: * *p* < 0.05, ** *p* < 0.01, *** *p* < 0.001 versus CON group; $ *p* < 0.05 versus DIA group; # *p* < 0.05 versus ACY group.

Figure 9A illustrates the outcomes of a one-way ANOVA, which revealed statistically significant variations in AChE levels across the experimental groups [F(3,20) = 11.57, *p* = 0.0001]. The AChE concentrations were quantified in ng/g of protein. Post hoc Tukey–Kramer analysis further identified specific group-wise differences. The DIA + ACY group exhibited notably elevated AChE levels compared to all other groups. Specifically, AChE levels in the DIA + ACY group (5.19 ± 0.76, *p* < 0.001) in the brain tissues were significantly higher than those observed in the CON group (1.72 ± 0.21), as well as the DIA (3.5 ± 0.21, *p* < 0.05) and ACY (3.5 ± 0.18, *p* < 0.05) groups. Both the DIA and ACY groups also demonstrated remarkable increases in AChE levels relative to the CON group. No additional statistically significant differences were observed among the groups.

Figure 9B presents BACE-1 results, where one-way ANOVA revealed significant differences among the groups [F(3,20) = 7.065, *p* = 0.002]. BACE-1 levels were assessed in ng/g of protein. Post hoc analysis confirmed that all treatment groups exhibited elevated BACE-1 levels compared to the CON group. Specifically, the DIA group (4.25 ± 0.43, *p* < 0.05), the ACY group (4.43 ± 0.49, *p* < 0.05), and the DIA + ACY group (5.03 ± 0.55, *p* < 0.01) all demonstrated significantly higher values than the CON group (2.18 ± 0.38). No other differences were observed across the remaining groups.

The one-way ANOVA identified significant differences among all experimental groups [F(3,20) = 13.66, *p* < 0.0001]. Figure 9C illustrates the outcomes for GSK-3β. GSK-3β concentrations were also documented in ng/g of protein. Tukey–Kramer post hoc comparisons indicated that GSK-3β levels were statistically elevated in all treated groups. Specifically, the DIA group (4.85 ± 0.41, *p* < 0.01), the ACY group (5.28 ± 0.42, *p* < 0.01), and the DIA + ACY group (6.4 ± 0.62, *p* < 0.001) demonstrated substantial increases relative to the CON group (2.58 ± 0.19). No other significant differences in GSK-3β levels were observed across the experimental groups.

### 3.7. Impact of DIA and ACY on Specific Neuroinflammatory Markers in the Rat Brain

**Figure 10:**
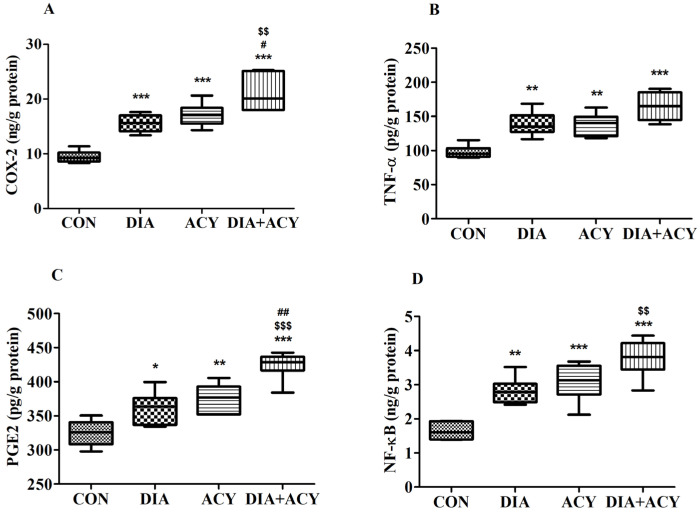
Effects of DIA and ACY exposure on inflammatory markers in rats: (**A**) COX-2, (**B**) TNF-α, (**C**) PGE2, and (**D**) NF-κB. Data are shown as mean ± SEM (n = 6 per group). One-way ANOVA with Tukey–Kramer post hoc test was used. Significance levels: * *p* < 0.05, ** *p* < 0.01, *** *p* < 0.001 versus CON group; $$ *p* < 0.01, $$$ *p* < 0.001 versus DIA group; # *p* < 0.05, ## *p* < 0.01 versus ACY group.

The analysis of COX-2 levels across the experimental groups revealed considerable changes, as determined by one-way ANOVA [F(3,20) = 29.55, *p* < 0.0001] (Figure 10A). The concentration of COX-2, measured in ng/g of protein, was further analyzed using Tukey–Kramer post hoc comparisons. The DIA + ACY group displayed the highest COX-2 levels (21.09 ± 1.35), exhibiting a statistically significant increase compared to the CON group (9.44 ± 0.45, *p* < 0.001), the DIA group (15.56 ± 0.65, *p* < 0.01), and the ACY group (17.13 ± 0.85, *p* < 0.05). Additionally, both the DIA and ACY groups showed elevated COX-2 levels (*p* < 0.001) relative to the CON group. The remaining groups did not display any further significant differences.

As illustrated in Figure 10B, levels of TNF-α exhibited significant variation among the treatment groups [F(3,20) = 15.83, *p* < 0.0001], measured in pg/g protein. The DIA group (138.7 ± 7.07, *p* < 0.01), ACY group (138.1 ± 6.65, *p* < 0.01), and DIA + ACY group (164.9 ± 9.29, *p* < 0.001) all demonstrated significant increases relative to the CON group (97.56 ± 3.87). No further differences in TNF-α levels were identified among the groups.

Similarly, the PGE2 results reflected the COX-2 pattern (Figure 10C). One-way ANOVA revealed substantial differences between groups [F(3,20) = 22.85, *p* < 0.0001]. PGE2 concentrations, expressed in pg/g protein, were notably higher in the DIA + ACY group (424.4 ± 8.4) than in the CON group (324.8 ± 7.81, *p* < 0.001), DIA group (361.2 ± 9.66, *p* < 0.001), and ACY group (375.5 ± 8.53, *p* < 0.01). Furthermore, both the DIA (*p* < 0.05) and ACY (*p* < 0.01) groups exhibited significantly increased PGE2 levels compared to the CON group. The remaining groups failed to demonstrate statistically significant differences in PGE2 levels.

Figure 10D displays the results for NF-κB levels, which also demonstrated significant variation [F(3,20) = 22.84, *p* < 0.0001] according to ANOVA. NF-κB levels, measured in ng/g protein, were markedly elevated in the DIA + ACY group (3.78 ± 0.22) relative to the CON group (1.647 ± 0.1, *p* < 0.001) and the DIA group (2.81 ± 0.16, *p* < 0.01). However, no statistically significant differences were observed between the DIA + ACY and ACY-only groups. Both the DIA (*p* < 0.01) and ACY (3.08 ± 0.23, *p* < 0.001) groups showed elevated NF-κB levels compared to the CON group. The other groups did not display any statistically significant differences in NF-κB levels.

### 3.8. Impact of DIA and ACY on Specific Apoptosis Markers in the Rat Brain

**Figure 11:**
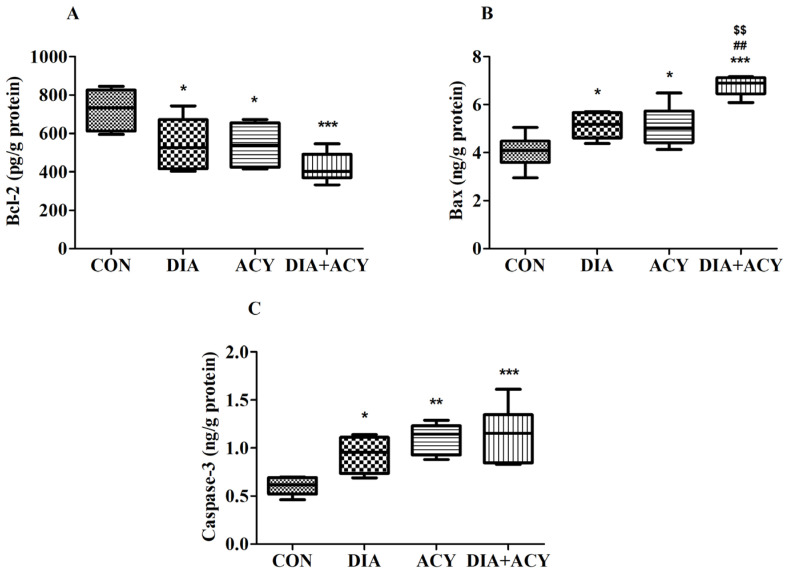
Effects of DIA and ACY administration on apoptosis-related markers in rat brain tissue: (**A**) Bcl-2, (**B**) Bax, and (**C**) Caspase-3. Data are shown as mean ± SEM (n = 6 per group). One-way ANOVA with Tukey–Kramer post hoc test was used. Significance levels: * *p* < 0.05, ** *p* < 0.01, *** *p* < 0.001 versus CON group; $$ *p* < 0.01 versus DIA group; ## *p* < 0.01 versus ACY group.

As illustrated in Figure 11A, the levels of Bcl-2 exhibited significant differences among the experimental groups as determined by a one-way ANOVA [F(3,20) = 7.627, *p* = 0.0014]. Measurements were expressed in pg/g of protein. Subsequent Tukey–Kramer post hoc analysis revealed that all treatment groups exhibited significant reductions in Bcl-2 levels compared to the CON group. Specifically, the DIA group (544.5 ± 56.42, *p* < 0.05), the ACY group (539.9 ± 44.96, *p* < 0.05), and the DIA + ACY group (422.9 ± 31.24, *p* < 0.001) each showed substantial decreases relative to the CON group (724.3 ± 43.95). No additional differences were observed among the remaining groups.

Figure 11B summarizes the results regarding Bax protein expression. A one-way ANOVA revealed significant differences between the groups [F(3,20) = 19.31, *p* < 0.0001]. Post hoc analyses confirmed that the DIA + ACY group exhibited the highest Bax levels across all groups. This group (6.79 ± 0.16) demonstrated a significant increase compared to the CON group (4.04 ± 0.28, *p* < 0.001), the DIA group (5.13 ± 0.22, *p* < 0.01), and the ACY group (5.1 ± 0.34, *p* < 0.01). Furthermore, Bax levels in the control group were significantly lower than those observed in all other treatment groups. No additional significant differences were observed among the remaining groups variations.

An ANOVA demonstrated a statistically significant increase in Caspase-3 levels across the various experimental conditions [F(3,20) = 9.105, *p* = 0.0001], as shown in Figure 11C. Caspase-3 concentrations were quantified in nanograms per gram of protein. The DIA (0.93 ± 0.08, *p* < 0.05), ACY (1.1 ± 0.07, *p* < 0.01), and DIA + ACY (1.14 ± 0.12, *p* < 0.001) groups all exhibited markedly elevated levels relative to the CON group (0.60 ± 0.04). No significant differences in Caspase-3 levels were observed in the remaining groups’ expression.

### 3.9. Impact of DIA and ACY on Specific Oxidative Markers in the Rat Brain

**Figure 12:**
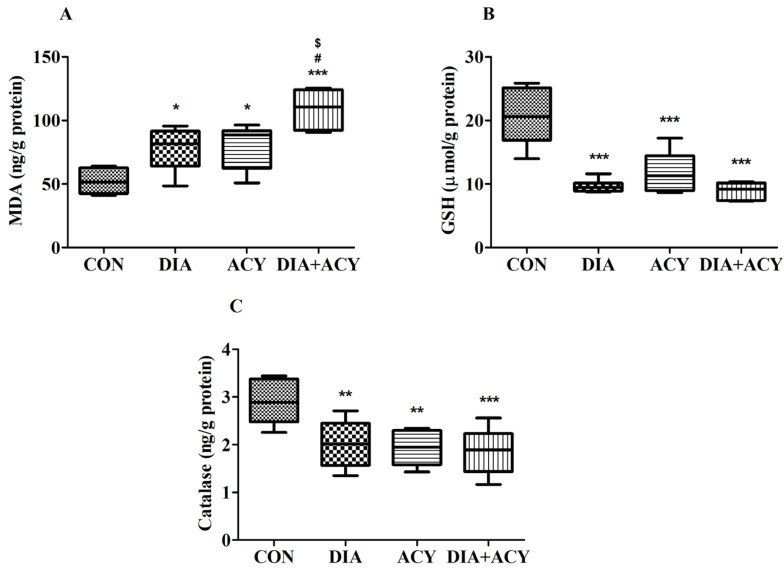
Effects of DIA and ACY administration on oxidative stress markers in rats: (**A**) MDA, (**B**) GSH, and (**C**) Catalase. Data are shown as mean ± SEM (n = 6 per group). One-way ANOVA with Tukey–Kramer post hoc test was used. Significance levels: * *p* < 0.05, ** *p* < 0.01, *** *p* < 0.001 versus CON group; $ *p* < 0.05 versus DIA group; # *p* < 0.05 versus ACY group.

As illustrated by Figure 12A, MDA levels exhibited statistically significant differences among the experimental groups, according to a one-way ANOVA test [F(3,20) = 13.58, *p* < 0.0001]. Tukey–Kramer post hoc analysis further elucidated intergroup differences. The MDA concentration, measured in ng/g protein, was highest in the DIA + ACY group (109 ± 6.44). This group demonstrated a marked elevation compared to the CON group (52.25 ± 4.17, *p* < 0.001), the DIA group (77.93 ± 6.91, *p* < 0.05), and the ACY group (80.19 ± 7.22, *p* < 0.05). Additionally, both DIA and ACY groups showed significantly increased MDA levels relative to the CON group. No statistically significant differences in MDA levels were observed among the remaining groups.

Figure 12B presents the findings for GSH. The analysis indicated substantial variation in GSH levels across groups [F(3,20) = 21.27, *p* < 0.0001]. These levels, measured in μmol/g protein, were significantly lower in all treatment groups than in the CON group. Specifically, GSH levels in the DIA group (9.64 ± 0.42, *p* < 0.001), ACY group (11.86 ± 1.32, *p* < 0.001), and DIA + ACY group (8.94 ± 0.54, *p* < 0.001) were markedly decreased relative to the CON group (20.64 ± 1.8). No further significant differences were identified among the treatment groups.

As shown in Figure 12C, catalase levels followed a pattern similar to that of GSH. ANOVA results indicated significant differences in catalase activity across all experimental groups [F(3,20) = 6.638, *p* = 0.0027]. Catalase values, recorded in ng/g protein, were significantly reduced in the DIA group (2.02 ± 0.21, *p* < 0.01), the ACY group (1.93 ± 0.16, *p* < 0.01), and the DIA + ACY group (1.86 ± 0.2, *p* < 0.001) when compared with the CON group (2.9 ± 0.19). No statistically significant variations in catalase levels were observed among the remaining groups.

### 3.10. Impact of DIA and ACY on Specific Immunohistochemistry Markers in the Rat Brain

**Figure 13:**
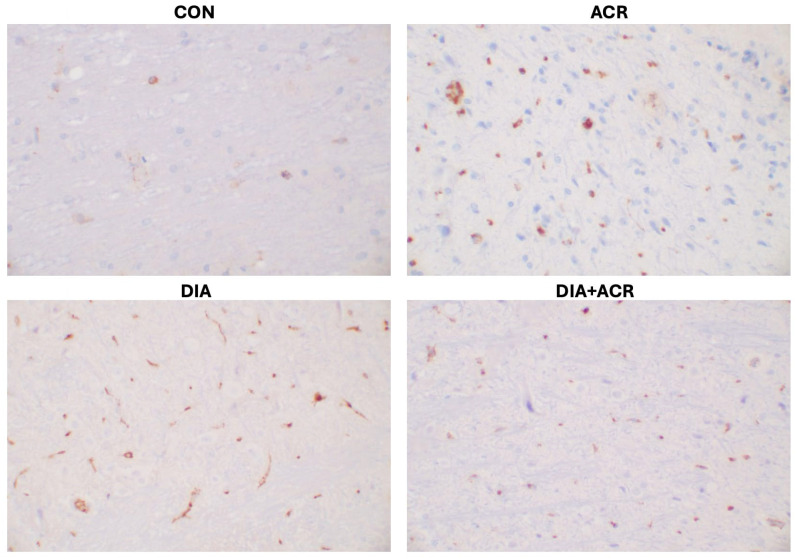
Photomicrographs of Bcl-2 immunohistochemical staining in rat cerebral tissue (Concentrated, Monoclonal antibody, Clone: BCL-2/100/D5; magnification ×400, captured using Motic BA410E microscope, Motic, Xiamen, China).

As illustrated in Figure 13, the CON group exhibited minimal Bcl-2 immunoreactivity, with only four positively stained cells observed. Conversely, the ACY group demonstrated the prominent number of Bcl-2-stained cells (41 cells) in the brain tissue, followed by the DIA group and DIA + ACY group, which showed comparable levels of expression, with 36 and 35 stained cells, respectively (Table 2). These findings suggest an intensified apoptotic response in all treatment groups relative to the CON group. Interestingly, as shown in Figure 11A, ELISA analysis demonstrated a significant reduction in overall Bcl-2 protein levels across all treatment groups compared to the CON group.

**Figure 14:**
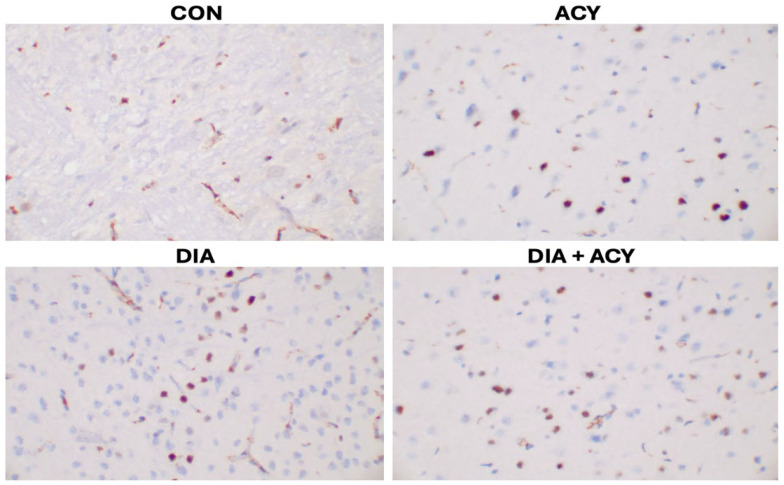
Photomicrographs of Bcl-6 immunohistochemical staining in rat cerebral tissue (Ready to Use, Monoclonal antibody, Clone: LN22; magnification ×400, captured using Motic BA410E microscope).

The CON group exhibited limited Bcl-6 expression, as Figure 14 illustrates, with only five cells displaying positive staining (Table 2). Conversely, the DIA + ACY group demonstrated the most prominent immunoreactivity among all experimental groups, with twenty-eight Bcl-6-positive cells. The DIA group showed moderate staining, with eighteen positive cells, whereas the ACY group presented a lower count of thirteen stained cells within the cerebral tissue. Collectively, these findings indicate an elevated neuroinflammatory response activity across the various treatment groups, with differing levels of intensity. Furthermore, the observed differences among the treatment groups, in comparison to the CON group, suggest that the extent of neuroinflammation varies according to the specific treatment administered groups.

**Figure 15:**
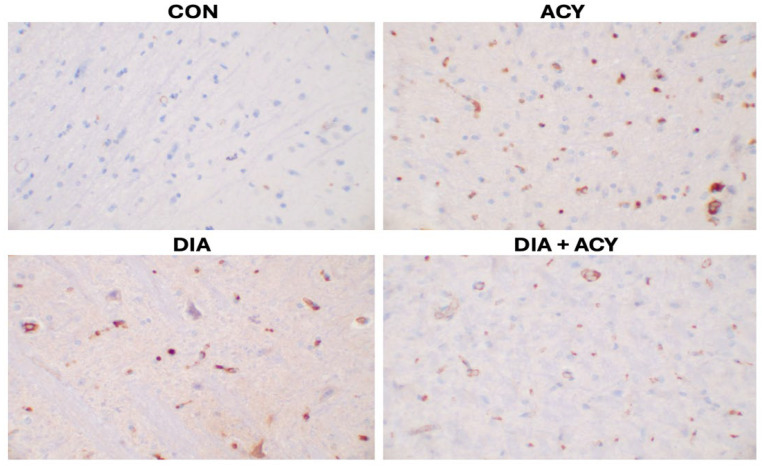
Photomicrographs of CD138 immunohistochemical staining in rat cerebral tissue (Ready to Use, Monoclonal antibody, Clone: MJ15; magnification ×400, captured using Motic BA410E microscope).

As illustrated in Figure 15, the CON group exhibited minimal immunoreactivity expression of CD138, with only one positively stained cell observed. Conversely, the ACY group demonstrated the most prominent immunoreactivity (38 cells) of CD138 in the rat cerebral tissue. Furthermore, the DIA + ACY group displayed the expression of 23 CD138-stained cells in rat cerebral tissue, while the DIA group showed 21 CD138-stained cells in rat cerebral tissue (Table 2). These findings indicate that the healing process was significantly activated across the treatment groups, with varying degrees of intensity. The differential responses observed among the treatment groups, as well as in comparison to the CON group, suggest distinct levels of tissue repair and recovery mechanisms.

**Figure 16:**
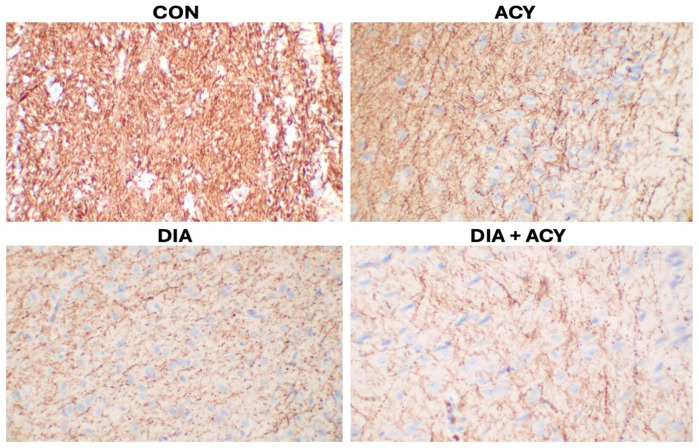
Photomicrographs of NF immunohistochemical staining in rat cerebral tissue (Ready to Use, Monoclonal antibody, Clone: 2F11; magnification ×400, captured using Motic BA410E microscope).

According to our findings (Figure 16), the staining intensity was markedly reduced in both the DIA group and the DIA + ACY group, indicating significant neuronal damage. Conversely, the ACY group demonstrated a milder reduction in staining, suggesting less severe injury. When compared to the CON group, all treatment groups showed diminished staining, indicating varying levels of neuronal impairment among the experimental cohort groups.

## 4. Discussion

**Figure 17:**
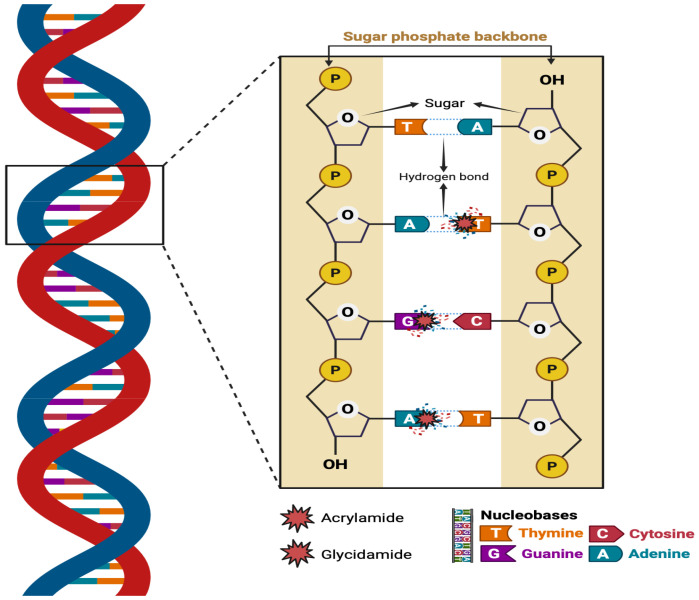
The reaction of acrylamide and glycidamide with DNA bases can lead to the formation of adducts. Created in BioRender. Alshammari, A. (2026). https://biorender.com/yqtdur3 (accessed on 6 February 2026).

Smoking and diet increase ACY exposure, contributing to T2DM through OS, inflammation, and insulin resistance [13,22]. ACY further exacerbates oxidative damage, neuroinflammation, and cognitive dysfunction in DM, with evidence linking it to elevated blood glucose and metabolic disturbances [9]. ACY is metabolized via two pathways: CYP2E1 converts it to glycidamide, causing DNA damage and neurotoxicity, while the other pathway mainly detoxifies it [14,15] (Figure 17). This study investigated the detrimental effects of ACY exposure in T2DM rats, induced by NTH and STZ. It aimed to understand how ACY impacts OS, neurodegeneration, neuroinflammation, and neuroapoptosis in brain homogenate tissue. Furthermore, the research assessed the effects of ACY on neurobehavioral performance, brain morphology, blood glucose levels, and overall health status and weight.

The selection of the ACY dose in this study was based on previous findings to ensure both efficacy and safety. The study reported that ACY at 50 mg/kg resulted in severe neurotoxicity and mortality in rats, while 25 mg/kg caused leg weakness after repeated dosing. In contrast, a lower dose of 10 mg/kg showed no detectable neurotoxic effects even after prolonged exposure [32]. Therefore, an intermediate dose of 20 mg/kg ACY was selected to induce measurable neurotoxic effects without causing excessive lethality. This dose has also been used in similar experimental neurotoxicity models and in our previous studies to investigate the mechanisms of ACY-induced neurotoxicity [14,24].

The initial cognitive assessment in this study was the EPM, administered over two consecutive days, beginning with the acquisition phase and subsequently the retention phase. This assessment evaluated learning and memory capabilities in the subjects, utilizing their innate preference for enclosed arms over open arms. A reduction in TL values was considered indicative of enhanced spatial memory [24]. On both the first and second days of EPM testing, the results demonstrated a consistent pattern across all groups. All treatment groups exhibited statistically significant differences versus the CON group. The DIA + ACY group exhibited a greater difference in outcomes compared to the other treatment groups, i.e., the DIA group and the ACY group. However, despite this observed increase, the difference in the DIA + ACY group did not reach statistical significance. In a comparable manner, dietary ACY worsens DM-related cognitive dysfunction, likely through oxidative damage, neuroinflammation, and metabolic disturbances [9]. Furthermore, studies have shown that mild cognitive impairment affects about 45% of individuals diagnosed with T2DM [7].

The second cognitive assessment conducted, following the EPM, was the NOR test, which was employed to assess the recognition memory capabilities of the rats. It was executed over two sequential days, comprising three distinct 5 min phases. On the initial day, the subjects underwent a habituation phase within an empty wooden box. On the subsequent day, the training and testing phases were carried out, separated by a four-hour interval. The duration of exploration towards the ETFO and ETNO was recorded, and their discriminatory ability was evaluated through the PDI [24]. In addition, studies involving substantial populations indicate that individuals with diabetes mellitus are at an increased risk of experiencing memory impairments and dementia. This finding raises significant concerns for healthcare professionals and public health institutions [6]. The findings from the ETFO test revealed a notable decline in performance across all treatment groups when compared with the CON group, supported by consistently significant *p*-values. Similarly, the ETNO test demonstrated a consistent pattern, with significant reductions observed across all treatment groups relative to the CON group. Notably, the DIA + ACY group exhibited a statistically significant difference in comparison to the DIA-only group, suggesting an additive or exacerbating effect. Regarding the DI%, the data revealed clear distinctions between each experimental group and the CON group; however, all treatment groups demonstrated similar effects without statistical significance variation.

On the final day of the experiment, the Y-maze test was conducted to assess the animals’ exploration of a novel environment and their proficiency in navigating an unfamiliar maze arm. The parameters evaluated included NEKA, NENA, and the respective TSNA% values. Recent studies have indicated that T2DM is linked to impairments in executive functions, working memory, psychomotor speed, and attention, thereby exacerbating cognitive deficits [33]. The results for NEKA, NENA, and TSNA% in this study, which employed the same ACY dosage, exposure duration, and route of administration, were predominantly in agreement with our previous findings, especially when compared to the CON group [14]. Furthermore, the DIA + ACY group demonstrated a more significant change in outcomes relative to the individual treatment groups (DIA and ACY alone); however, this improvement did not attain statistical significance.

Regarding neurodegenerative markers, multiple studies have demonstrated that AChE plays a pivotal role in the progression of neurodegenerative disorders by regulating inflammatory pathways, facilitating neuronal cell death, increasing OS, and contributing to the accumulation of abnormal protein aggregates [34]. Furthermore, prior research has demonstrated that elevated plasma activity and concentrations of BACE1 are significantly associated with increased amyloid-β deposition in the brain and long-term neurobehavioral performance. These findings underscore the potential of plasma BACE1 as a valuable biomarker for predicting the onset and progression of Alzheimer’s disease [35]. Furthermore, GSK-3β is vital to numerous cerebral processes, including the maintenance of neuronal structure, synaptic plasticity, neuroinflammatory signaling, and the pathogenic mechanisms underlying various neurological diseases [36]. Moreover, a strong correlation has been observed between GSK-3 and the pathogenesis and progression of several chronic diseases like DM, obesity, and various forms of cancer [37]. On this basis, the current study considers AChE, BACE1, and GSK-3β as key biomarkers of neurodegeneration. The levels of all treatment groups were significantly higher than those in the CON group. Notably, AChE levels were markedly elevated in the treatment group receiving DIA and ACY, surpassing the levels observed with either treatment alone, thereby indicating a potential exacerbating effect on cholinergic function impairment.

Neuroinflammation represents a defensive response within the brain that is initiated to protect the CNS from both internal and external damage. When inflammatory mediators are released excessively or are chronically activated, the response can become detrimental, resulting in structural damage to the CNS as well as impairing its physiological functions [38]. In the present study, COX-2, TNF-α, PGE2, and NF-κB were identified as primary indicators of neuroinflammatory activity. Cyclooxygenase enzymes, COX-1 and COX-2, are constitutively expressed within the CNS, where they contribute to the regulation of inflammatory processes. Notably, COX-2 is the more predominantly expressed isoform in the brain and spinal cord and is particularly associated with neuroinflammatory responses under pathological conditions [39].

The PGE2 and COX signaling cascades have also gained increasing prominence as primary therapeutic targets in the development of anti-inflammatory interventions for neurodegenerative diseases [40]. The pro-inflammatory cytokine TNF-α is primarily produced by glial cells and neurons, including astrocytes and microglia, which constitute the main glial cell population involved in neuroinflammatory responses [41]. Through the activation of inflammatory cytokine signaling pathways, TNF-α serves as a principal regulator of the acute-phase inflammatory response and plays a significant role in perpetuating chronic inflammation [42]. Furthermore, TNF-α is involved in the maturation of dendritic cells, synaptic connectivity, modulation of neuronal responsiveness to various sensory stimuli, and regulation of homeostatic plasticity within the CNS [38,43]. NF-κB is of particular significance in the control of neuroinflammatory and neurodegenerative processes and is of increasing interest as a promising therapeutic target for the treatment of various neuro diseases [44]. Unsurprisingly, all treatment groups demonstrated significantly elevated levels of COX-2, TNF-α, PGE2, and NF-κB compared to the CON group. Notably, TNF-α levels were elevated across all treatment groups, although no significant differences were observed among these groups concerning this biomarker. Conversely, the combination of DIA + ACY markedly increased the levels of COX-2 and PGE2 relative to the individual treatments of DIA and ACY, achieving peak levels within all experimental conditions. Regarding NF-κB, an increase was observed to be greater in the DIA + ACY group compared to the DIA group; however, this difference was not statistically significant when compared to the ACY group isolation.

To investigate the effect of ACY exposure on T2DM concerning apoptotic signaling in brain tissue, the study examined the expression levels of the principal apoptotic regulators: Bcl-2, Bax, and Caspase-3. Apoptosis, a highly regulated and intrinsic cellular process, is characterized by the coordinated disassembly of cells and plays a critical role in cellular homeostasis. Through the elimination of damaged or unwanted cells, apoptosis contributes to normal development and maintains tissue homeostasis within multicellular organisms [45]. It occurs during normal physiological processes and as a result of cellular stress. Apoptosis may proceed through two principal pathways: the extrinsic pathway, which is triggered by signals that activate external death receptors on the cell surface, and the intrinsic pathway, which is initiated by internal cellular stress and mitochondrial damage. Although each pathway is initiated by distinct mechanisms, they ultimately converge at a common point to execute the process of programmed cell death [46].

OS assumes a crucial role in the advancement of neurodegenerative diseases by promoting apoptotic cell death, predominantly through the overproduction of reactive oxygen species (ROS), which impair mitochondrial function. Within the intrinsic pathway, Bax facilitates the release of cytochrome c from mitochondria, thereby initiating a cascade that activates Caspase-3 and leads to DNA fragmentation and cell death. Conversely, Bcl-2 functions as an anti-apoptotic protein, counteracting this process by maintaining mitochondrial integrity. In the extrinsic pathway, tumor necrosis factor-alpha (TNF-α) activates death receptors, which subsequently activate Caspase-8 and Caspase-3, thereby promoting apoptosis [47].

In the current study, all apoptotic biomarkers exhibited significant alterations in all treatment groups versus the CON group. No additional meaningful changes were found between the treatment groups, except for Bax levels, where the DIA + ACY group demonstrated a markedly higher increase than all other experimental groups, including the DIA group and the ACY group individually. An intriguing discrepancy was observed between our immunohistochemistry and ELISA results regarding Bcl-2 expression. Immunohistochemistry showed a significant increase in Bcl-2–positive cells across all treatment groups compared to the control, whereas ELISA indicated a notable reduction in overall Bcl-2 protein levels within the same groups. Several biological and technical factors might explain this divergence. First, Bcl-2 expression in the brain is inherently heterogeneous and varies among different cell types and regions, reflecting its tightly regulated, context-dependent role in neuronal survival [48]. Under stress conditions, subsets of neurons or glial cells may upregulate Bcl-2 as a protective, compensatory response, leading to an apparent increase in the number of immunohistochemically positive cells in specific areas. However, when measured in whole-tissue homogenates, the total Bcl-2 protein content may still decrease, likely due to neuronal loss or downregulation in more vulnerable populations [49].

Second, immunohistochemistry primarily identifies the proportion of cells surpassing a positivity threshold, providing valuable spatial information but not quantifying staining intensity or absolute protein content per cell [50]. Consequently, numerous weakly positive cells may inflate cell counts without corresponding to a net increase in the tissue-wide Bcl-2 content. Thirdly, regional sampling differences between immunohistochemistry (limited to selected brain regions) and ELISA (whole-tissue homogenates) may also contribute to the contrasting results, particularly if Bcl-2 expression varies across different regions. Finally, methodological factors such as differences in antibody epitopes, tissue fixation, and protein extraction efficiency could further influence detection accuracy.

Collectively, these findings emphasize the importance of integrating histological and biochemical methodologies, as they offer complementary insights: immunohistochemistry elucidates the spatial and cellular distribution of Bcl-2 expression, while ELISA quantifies overall protein abundance. The observed inconsistency, therefore, does not compromise the validity of the data but rather highlights the complex regulation of Bcl-2 in response to DIA and ACY exposure.

Regarding oxidative markers, OS arises when an imbalance between the generation of ROS and the efficiency of the cellular antioxidant defense mechanisms leads to excessive ROS accumulation [51]. Superoxide anion, a major cellular ROS, is produced during mitochondrial oxidative phosphorylation and by NADPH oxidase at the plasma membrane [52,53]. It is detoxified by enzymatic antioxidants like SOD, CAT, and GPX, and by non-enzymatic antioxidants such as GSH. Proper regulation of ROS levels is crucial for neurodevelopment, function, and survival [54,55]. When this process takes place in brain cells, it can impair essential neuronal functions, including memory formation, learning ability, and cognitive performance [56]. In this study, the lipid peroxidation marker MDA was significantly elevated across all treatment groups compared to the CON group, with the DIA + ACY group exhibiting the most pronounced increase among all experimental conditions. Conversely, the antioxidant markers GSH and catalase were significantly reduced in all treatment groups relative to the CON group, but with no significant differences observed between the treatment groups themselves. These findings indicate a state of pronounced oxidative imbalance, particularly intensified in the combined DM and ACY group.

For immunohistochemical biomarkers, in addition to Bcl-2, this study also evaluated the expression of Bcl-6, CD138, and neurofilaments (NF) in cerebral tissue of rats to assess the ramifications of ACY exposure in T2DM. Bcl-6 is a transcriptional repressor, though, and it regulates the expression of genes involved in DNA repair, cell cycle, and cell differentiation. It is also important in regulating immune responses and integrating inflammatory signaling pathways [57]. Another marker, CD138, is a transmembrane protein that primarily occurs in plasma cells [58]. It performs different functions, including adhesion of cells, repair of wounds, regulation of cell growth, and differentiation of different cells, but mainly plasma cells [59]. Further, NF is an intermediate filament protein that exists mainly in the cytoplasm of neurons [60]. NFs are prevalent cytoskeletal structures in axons, playing a vital role in ensuring structural stability. They both move bidirectionally along microtubule paths, where they serve a role in the regulation of axonal diameter and ensure the efficient intracellular transport dynamics required for normal neuronal operation [61]. This protein is usually lost during any injury to the neurons.

The DIA + ACY group demonstrated the highest expression of Bcl-6 protein among all experimental groups. This may indicate increased neuroinflammation in this group compared to all experimental groups. In addition, the ACY group showed the highest CD138 expression in the brain, indicating a strong repair response to ACY-induced damage. In contrast, the DIA and DIA + ACY groups had lower CD138 levels, suggesting that DM impairs the brain’s ability to initiate proper healing, as chronic hyperglycemia disrupts cellular repair mechanisms, suppresses oligodendrogenesis, and impairs microglial-mediated regeneration—all processes that are essential for function and structural recovery in the brain [62].

Similar to CD138 expression, NF staining revealed no neuropathological changes in the brain tissue of the CON group compared to all treatment groups. Notably, the ACY group exhibited the lowest NF staining intensity, indicating a pronounced neurotoxic effect. In contrast, both the DIA and combined DIA + ACY groups showed comparable levels of NF staining, which were higher than in the ACY group, suggesting that while neurodegeneration occurred, it was less severe than in the ACY group.

The present study provides an integrated neurobehavioral and molecular evaluation of ACY exposure in a T2DM rat model; however, several limitations should be considered. Although multiple biomarkers related to neurodegeneration, oxidative stress, neuroinflammation, and apoptosis were assessed, the number of markers representing each pathway was limited. In addition, biochemical analyses were performed using whole-brain homogenates, which may mask regional variations in neuronal vulnerability. The study also included only male rats to reduce biological variability, and future investigations should incorporate both sexes to better understand potential sex-related differences. Finally, the experimental exposure period was relatively short and may not fully reflect the long-term effects of chronic T2DM and ACY exposure. Future studies should therefore explore longer exposure durations and include more detailed molecular analyses to further clarify the mechanisms underlying ACY-induced neurotoxicity in T2DM.

## 5. Conclusions

The present study provides comprehensive evidence that smoking and dietary exposure to ACY exacerbate cognitive dysfunction, OS, neuroinflammation, neurodegeneration, brain structural damage, and neuroapoptosis, thereby augmenting neurotoxicity in a rat model of T2DM. Exposure to ACY in rats, particularly within diabetic groups, resulted in significant impairments in cognitive performance across multiple behavioral assessments, namely the EPM, NOR, and Y-Maze tests. At a molecular level, ACY exposure led to markedly elevated levels of neurodegenerative biomarkers, including AChE, BACE1, and GSK-3β, indicating its role in fostering neuropathological alterations. Additionally, neuroinflammatory mediators such as COX-2, PGE2, TNF-α, and NF-κB were significantly increased, with the highest levels observed in the DIA + ACY group, suggesting an amplification of inflammation in diabetic neuropathology. Analysis of apoptotic pathways revealed notable dysregulation of both pro-apoptotic markers, Bax and Caspase-3, and the anti-apoptotic marker Bcl-2, with Bax expression being notably elevated in the DIA + ACY group. Furthermore, assessments of oxidative stress revealed a substantial increase in MDA levels, alongside a significant decline in antioxidant defenses, such as GSH and catalase, across all treatment groups, particularly in the DIA + ACY group. Immunohistochemical analyses evaluating Bcl-2, Bcl-6, CD138, and NF demonstrated structural alterations within the brain tissue. Overall, this study underscores the deleterious effects of ACY exposure on neuronal integrity and cognitive function in T2DM rats. It highlights the necessity of public health initiatives to mitigate dietary and smoking-related intake of ACY, especially among individuals with T2DM. Future research should explore the long-term consequences of ACY exposure, elucidate its detailed molecular mechanisms, and investigate potential neuroprotective strategies.

## Figures and Tables

**Table 1:** Immunohistochemical antibodies employed to stain brain tissue were selected based on their specificity to targeted neuropathological processes: BCL-2 as an indicator of apoptosis, BCL-6 to signify inflammation, CD138 to reflect immune cell activity, and neurofilaments (NF) to depict neuronal structural components integrity.

Stain	Manufacturer	Formulation	Antibody Type	Clone	Target
BCL-2	Leica Biosystems (Buffalo Grove, IL, USA)	Concentrated	Monoclonal	BCL-2/100/D5	Apoptosis
BCL-6	Leica Biosystems (Buffalo Grove, IL, USA)	Ready to Use	Monoclonal	LN22	Inflammation
CD138	Agilent Dako (Santa Clara, CA, USA)	Ready to Use	Monoclonal	MJ15	Immune Activity
NF	Agilent Dako (Santa Clara, CA, USA)	Ready to Use	Monoclonal	2F11	Neuronal Structural

**Table 2:** All quantitative evaluations of stained slides were conducted at a magnification of ×400 utilizing the Motic BA410E microscope, concentrating on the most immunoreactive (hotspot) regions within each section. Results are presented as the count of positively stained cells per high-power field. Staining was deemed positive when cytoplasmic coloration was evident, except for BCL-6, for which only nuclear staining was evaluated.

Stain	CON Group	ACY Group	DIA Group	DIA + ACY Group
BCL-2	4	41	36	35
BCL-6	5	13	18	28
CD138	1	38	21	23

## Data Availability

The data presented in this study are available in the article; further inquiries can be directed to the corresponding authors.

## Abbreviations

The following abbreviations are used in this manuscript:

ACY	Acrylamide
AChE	Acetylcholinesterase
ARRIVE	Animal Research: Reporting of In Vivo Experiments
BACE1	β-Site Amyloid Precursor Protein Cleaving Enzyme-1
Bax	BCL-2–Associated X Protein
Bcl-2	B-Cell Lymphoma-2
Bcl-6	B-Cell Lymphoma-6
CD138	Cluster of Differentiation 138
CON	Control Group
COX-2	Cyclooxygenase-2
DIA	Diabetic Group
DI%	Discrimination Index Percentage
EPM	Elevated Plus Maze
ETFO	Exploration Time of Familiar Object
ETNO	Exploration Time of Novel Object
GSH	Reduced Glutathione
GSK-3β	Glycogen Synthase Kinase-3 Beta
IHC	Immunohistochemistry
MDA	Malondialdehyde
NENA	Number of Entries into Novel Arm
NEKA	Number of Entries into Known Arm
NF	Neurofilament
NF-κB	Nuclear Factor Kappa-B
NOR	Novel Object Recognition
NTH	Nicotinamide Hydrochloride
PGE2	Prostaglandin E2
ROS	Reactive Oxygen Species
STZ	Streptozotocin
T2DM	Type 2 Diabetes Mellitus
TL	Transfer Latency
TNF-α	Tumor Necrosis Factor-Alpha
TSNA%	Time Spent in Novel Arm Percentage
